# Survey of Commercial Food Products for Detection of Walnut (*Juglans regia*) by Two ELISA Methods and Real Time PCR

**DOI:** 10.3390/foods10020440

**Published:** 2021-02-17

**Authors:** Raquel Madrid, Aina García-García, Pablo Cabrera, Isabel González, Rosario Martín, Teresa García

**Affiliations:** Departamento de Nutrición y Ciencia de los Alimentos, Facultad de Veterinaria, Universidad Complutense de Madrid, 28040 Madrid, Spain; raqmad01@ucm.es (R.M.); ainagarcia@ucm.es (A.G.-G.); pablocabrera@gmail.com (P.C.); gonzalzi@vet.ucm.es (I.G.); rmartins@vet.ucm.es (R.M.)

**Keywords:** ELISA, food allergen labeling, food analysis, polyclonal antibody, real time PCR, ScFv recombinant antibody, walnut detection

## Abstract

Labeling of food allergens in accordance with legal regulations is important to protect the health of allergic consumers. The requirements for detecting allergens in foods involve adequate specificity and sensitivity to identify very small amounts of the target allergens in complex food matrices and processed foods. In this work, one hundred commercial samples were analyzed for walnut detection using three different methods: a sandwich enzyme-linked immunosorbent assay (ELISA) kit based on polyclonal antibodies, a direct ELISA using a recombinant multimeric scFv, and a real time PCR. The most sensitive method was real time PCR followed by sandwich ELISA kit and multimeric scFv ELISA. There was agreement between the three methods for walnut detection in commercial products, except for some heat-treated samples or those that contained pecan. The walnut ELISA kit was less affected by sample processing than was the multimeric scFv ELISA, but there was cross-reactivity with pecan, producing some false positives that must be confirmed by real time PCR. According to the results obtained, 7.0 to 12.6% of samples (depending on the analytical method) contained walnut but did not declare it, confirming there is a risk for allergic consumers. Moreover, there was one sample (3.7%) labelled as containing walnut but that tested negative for this tree nut. Genetic and immunoenzymatic techniques offer complementary approaches to develop a reliable verification for walnut allergen labeling.

## 1. Introduction

Regular consumption of walnuts is increasing because they are associated with beneficial effects on human health, providing protection against the development of cardiovascular-related diseases, age-related neurological disorders, and even some cancer types [1]. Walnut (*Juglans regia* L.) is eaten raw or used for the manufacture of sauces, yoghurts, sweets, oils, beverages, and as an ingredient to improve the quality of food products. 

On the other hand, the presence of walnut as a hidden allergen in many processed foods constitutes a serious risk for developing adverse reactions in allergic patients, from oral allergy symptoms to anaphylaxis [2]. Sensitization to walnut storage proteins is primarily acquired during childhood, although it can also be acquired at a later age. Moreover, in certain populations, IgE levels to storage proteins Jug r 1, Jug r 4, and vicilin fractions, but not to nonspecific lipid transfer proteins and PR-10 proteins, correlate with systemic reactions to walnut [3]. The prevalence of walnut allergy in Europe (Ig E sensitization to Jug r 4 and clinical reaction) has been reported as between 0.1 and 0.35%, with the highest percentage observed in Madrid [4]. The minimum amount of walnut protein that elicits an allergic reaction in 5% of the sensitized population is estimated to be 3–4 mg [2]. Nevertheless, individual variability of reaction thresholds to food allergens is high [5]. 

Walnut belongs to one (nuts) of the 14 allergenic foods that must be declared in food labelling in the European Union [6]. Enzyme-linked immunosorbent assays (ELISA) are currently used for the detection and quantification of allergenic proteins present in commercial products, and several ELISA kits are available for walnut detection with different sensitivities and cross-reactions [7]. In addition to immunoassays, highly sensitive polymerase chain reaction (PCR) techniques using walnut-specific primers are also available for the detection and quantification of walnut in food products [8,9]. Recently, there have been many cutting-edge advances that improve the sensitivity of allergen detection and quantification and take into account different food matrices as well as the effect of food processing, such as biosensors and MS methods [10]. However, when using MS-based methods to analyze allergens in food, the high cost of their platform, maintenance costs, and the need for professionals must be considered [11]. Improvement of such methods, together with the establishment of eliciting doses in the sensitized population, will allow more reliable verification of labelling compliance of food products, avoiding excessive precautionary allergen labelling (PAL) that produces a loss of confidence of the allergic consumers, and increasing the number of safe products at their disposal [12,13].

In this work we compared three different methodologies for walnut detection in food products (a direct ELISA using a recombinant antibody, a commercial sandwich ELISA kit, and a walnut real time PCR method) to consider their applicability for labelling verification regarding the detection of walnut allergens in commercial food products. 

## 2. Materials and Methods 

### 2.1. Materials and Chemicals

TBS (Tris-buffered saline) composition was 0.05 M Tris-HCl and 150 mM NaCl, pH 7.6. TBST was TBS containing 0.05% Tween 20. The protein extraction buffer consisted of 0.035 M phosphate solution containing 1 M NaCl, pH 7.5. Sample buffer was 0.5 M Tris-HCl buffer, pH 6.8, 10% SDS, 20% glycerol, 0.5% bromophenol blue as the tracking dye, and 5% β-mercaptoethanol. Transfer buffer consisted of 0.025 mol L^−1^ Tris, pH 8.3, 0.192 mol L^−1^ glycine, and 200 mL L^−1^ methanol. Unless otherwise stated, chemicals were provided by Sigma (Darmstadt, Germany).

The AlerTox^®^ Walnut ELISA kit (KT-5909, Biomedal Diagnostics, Sevilla, Spain) included the following components: 8 well separable strips coated with walnut specific polyclonal antibodies, walnut standards (50, 15, 5, 2, and 0 mg kg^−1^), conjugate solution, substrate solution Tetramethylbenzidine (TMB), stop solution (contains H_2_SO_4_), sample extraction and dilution buffer, and washing solution (compositions not provided).

### 2.2. Experimental Binary Mixtures

Four different ground walnut products were used as reference materials: a) raw peeled walnut (RPW), consisting of walnuts finely ground (IKA^®^ A11 analytical mill, Staufen, Germany) after the removal the thin soft and edible seed coat (testa); b) toasted peeled walnut (TPW), consisting of RPW subjected to baking at 160 °C for 13 min (Heraeus, Hanau, Germany); c) raw unpeeled walnut (RUPW), consisting of walnuts with *testa*, finely ground; and d) toasted unpeeled walnut (TUPW), consisting of RUPW baked at 160 °C for 13 min. To prepare the RPW reference material, the shelled walnuts were placed in a water bath at 90 °C for 1 min, and then quickly transferred to an iced water bath for another minute before pulling the skin off with sterile forceps.

To evaluate the sensitivity of the assay, and to use as reference samples, binary mixtures of corn flour and ground walnut were prepared using a mill (IKA A11). Four binary mixtures were prepared, containing from 10^5^ to 1 mg kg^−1^ of RPW, TPW, RUPW, or TUPW in corn flour. A concentration of 10^5^ mg kg^−1^ was prepared by adding 3 g of ground walnuts to 27 g of corn flour. Concentrations of 10^4^ to 1 mg kg^−1^ were made in a similar way with the previous mixtures. All the mixtures were stored in screw capped vials at −20 °C.

### 2.3. Heterologous Species and Commercial Products

A wide variety of tree nuts, vegetal and animal species, and commercial food products were purchased at different local stores and markets in Madrid (Spain). Commercial walnuts from Spain and California and pecans from USA and Mexico were considered for analysis. All food samples (50 g) and heterologous species (5 g) were finely ground using an IKA A11 analytical mill and stored in screw capped vials at −20 °C. 

### 2.4. Protein and DNA Extraction

Protein extracts from food samples to be analyzed with a commercial sandwich ELISA kit (Biomedal Diagnostics, Sevilla, Spain) were obtained according to the method recommended by the supplier. Briefly, 0.5 g of the ground sample were mixed with 10 mL of kit extraction buffer, incubated for 15 min in a water bath at 60 °C with vortex mixing every two minutes, centrifuged for 10 min at 2000× *g*, and the supernatant was filtered through a 0.45 µm syringe filter (Sartorius, Göttingen, Germany). The test was carried out according to the manufacturer’s instructions with minor variations.

For multimeric scFv ELISA analysis, each food sample (0.2 g) was mixed with 1.2 mL of protein extraction buffer, and the mixture was shaken for 10 min at room temperature in a vertical rotator (HulaMixer Sample Mixer, Invitrogen, Carlsbad, TX, USA) to extract soluble proteins. The slurry was centrifuged at 10,000× *g* for 10 min at 4 °C, and the supernatant was filtered through a 0.45 µm syringe filter (Sartorius, Göttingen, Germany). 

DNA extraction and purification for real time PCR analysis of samples was performed as previously described [9]. The DNA obtained from Wizard DNA Clean-up System kit (Promega, Madison, WI, USA) was eluted in 50 µL of sterile deionized water, and DNA concentration was measured with a NanoDrop ND-1000 spectrophotometer (NanoDrop Technologies Inc., Montchanin, Denmark). 

A negative control, without sample, was included in every protein or DNA extraction. All protein and DNA extracts were stored at −20 °C.

### 2.5. Sandwich ELISA Kit for Detection of Walnut in Food Samples

The test was carried out according to the manufacturer’s instructions with minor variations (Biomedal Diagnostics, Sevilla, Spain). One hundred microliters of undiluted or diluted (1:5, 1:10, 1:25 in protein extraction buffer) sample extract or controls were added to the wells of the provided coated plate for walnut specific antibody. After 20 min incubation at room temperature (25 °C), the wells were washed ten times with 200 μL of 1X washing solution. Then, 100 µL of conjugate solution was added to the wells, incubated for 20 min at room temperature, and washed again. Following 20 min incubation with 100 μL of substrate solution, 100 μL of stop solution were added, and the yellow signal was measured at 450 nm in a spectrophotometer (FLUOstar Optima, BMG Labtech, Ortenberg, Germany). A negative extraction control (without sample) and the 0 mg kg^−1^ standard were included in all trials, and each sample was analyzed in duplicate. The limit of detection (LOD) and limit of quantification (LOQ) were calculated as the concentration of the target protein that presents an absorbance value higher than the blank (corn flour) plus 3 or 10 times, respectively, its standard deviation [14]. A concentration–response curve using the standards provided by the kit was generated by plotting the absorbance values vs. the walnut concentration. The standard curve obtained was fitted to the four-parameter logistic equation using Origin 8.0 software (OriginLab Corp., Northampton, MA, USA) and compared with curves obtained plotting the RPW, TPW, RUPW, and TUPW absorbances undiluted or diluted 1:5 or 1:25 with protein extraction buffer. Cross-reactivity of the assay was calculated for eight tree nuts and soybean, as the amount of walnut estimated by interpolating the absorbance values of undiluted extracts in the logistic equation obtained with the kit calibrants.

### 2.6. Direct ELISA with Multimeric scFv

A direct ELISA using walnut-specific multimerized JrBSF-scFv (*Juglans regia* Biotinylated Soluble Fragment-single chain antibody, multimerized with ExtrAvidin-HRP) was also used to detect walnut protein [15]. 

The wells of microtiter plates (F96 MaxiSorp Nunc immunoplates, Thermo Fisher Scientific, Waltham, MA, USA) were coated for 16 h at 4 °C with 100 µL of the protein extracts diluted 1:100 in PBS. After washing three times with TBS, they were blocked with 200 μL of 3% bovine serum albumin (BSA) in TBS for 1 h at 37 °C and washed again. One hundred microliters of multimerized scFv (2 mg mL^−1^) diluted 1:500 (*v*/*v*) in TBST containing 1% BSA was added to each well, and the plates were shaken for 2 h at room temperature and washed again. Then, 100 μL TMB substrate solution was added to each well, and the plates were incubated with shaking for 10 min before addition of 50 μL 1 M sulfuric acid and measurement of Absorbance at 450 nm. All experiments were performed in duplicate on three different days. Three different concentrations of walnut in corn flour (10^5^, 10^3^, and 0 mg kg^−1^) were included in each plate as internal controls. A commercial product was considered positive for walnut when its absorbance value was higher than that of the 10^3^ mg kg^−1^ standard.

### 2.7. Real time PCR Analysis

Real time PCR analysis of commercial samples was performed using walnut specific primers (WalITSdir/WalITSinv) and probe (WalITSP) or pecan specific primers (PecITSdir/PecITSinv) and probe (PecITSP) as described in López-Calleja et al. [9] and Table 1. All real time PCR analyses were carried out in duplicate for each DNA extract, together with their corresponding 18S endogenous control. The use of this endogenous control is important to normalize RT-PCR results, because the 18 S rRNA gene has a very stable expression level [16].

### 2.8. SDS-PAGE and Western Blotting Analysis 

Sodium dodecyl sulphate polyacrylamide gel electrophoresis (SDS-PAGE) [17], was performed using polyacrylamide gels (12% resolving gel and 4% stacking gel) in a Mini-Protean Tetra Cell (Bio-Rad, Hercules, CA, USA) at 90 V for 45 min. After electrophoresis, one third of the gel was stained with Blue safe (NZytech, Lisbon, Portugal) and the other two-thirds were transferred into methanol-activated polyvinylidene difluoride (PVDF) membranes (Immuno-Blot PVDF membranes; Bio-Rad, Hercules, CA, USA) at 400 mA for 45 min using a Mini Trans-Blot Cell (Bio-Rad). The membranes were then blocked with 3% BSA in TBS for 1 h at 37 °C, washed three times with TBS, and incubated overnight at 4 °C with walnut specific JrBSF scFv diluted 1:1000 in TBST containing 1% BSA (TBST-BSA). After washing three times with TBS, one of the membranes was incubated for 2 h at 37 °C with Horseradish Peroxidase (HRP) conjugated anti-6X His tag^®^ antibody (ab1187) (AbCam plc, Cambridge, UK) diluted 1:5000 in TBST-BSA, washed three times with TBST, and revealed with the chemiluminescent substrate Clarity Western ECL (Bio-Rad). The other membrane was incubated with 1:5000 dilution of anti-c-Myc monoclonal antibody (9E10) (Thermo Fisher Scientific, Waltham, MA, USA), followed by 1:20,000 dilution of alkaline phosphatase (AP) conjugated anti-mouse IgG (Sigma, Darmstadt, Germany) in TBST-BSA, for 1 h at 37 °C, then washed three times with TBST and revealed with AP chromogenic substrate (Thermo Fisher Scientific). The Western blotting membranes were scanned using a ChemiDoc XRS system (Bio-Rad) to visualize bands.

### 2.9. Protein Identification

Bands of interest from the Blue Safe stained SDS-PAGE gel were cut out with a sterile scalpel and immersed in 5% acetic acid solution. Proteins were then gel reduced, alkylated, and digested with trypsin according to Sechi and Chait [18]. Analysis of peptides from protein digestion was performed using the 4800 Plus MALDI TOF/TOF Analyzer mass spectrometer (Applied Biosystems, MDS Sciex, Toronto, ON, Canada), at the Proteomic Unit of Complutense University of Madrid (Spain). Peptide mass fingerprint and some peptide fragmentation spectra were combined and searched for in the MASCOT v2.3 search engine (http://www.matrixscience.com) through the Global protein Server (Applied Biosystems) against the NCBI database (17,919,084 sequences; 6,150,218,869 residues) without taxonomy restriction or search parameters: carbamidomethylcysteine was used as the fixed modification and oxidized methionine was used as the variable modification; peptide mass tolerance 80 ppm; 1 missed trypsin cleavage site allowed, and MS/MS fragments tolerance, 0.3 Da.

The applied probability filter was that set by the search engine software itself, in this case MASCOT, which uses its own probability algorithm. Thus, in all protein identification, the probability scores were greater than the score fixed by MASCOT as significant with a *p*-value lower than 0.05.

## 3. Results and Discussion

### 3.1. Evaluation of the Sandwich ELISA Kit for Detection of Walnut

The Walnut ELISA kit (Biomedal) is a sandwich-type immunosorbent assay designed for the detection and quantification of walnut in food products using specific polyclonal antibodies. 

According to the supplier, this kit does not show cross-reactions against 30 food matrices. To verify specificity, eight matrices were analyzed in this work, and the absorbance values obtained were interpolated in the logistic equation (*y* = *A*_2_ + (*A*_1_ − *A*_2_)/(1 + (*x*/*x*_0_)^*p*^) where *A*_1_ is the minimum absorbance for no analyte (background signal), *p* is the curve slope at the inflection point, *x*_0_ is the *x* value at the inflection point, and *A*_2_ is the maximum absorbance at infinite concentration; being *A*_1_ = 0.01445, *A*_2_ = 2.02251, *x*_0_ = 9.72293, *p* = 1.35762, and *R*^2^ = 0.9949, obtained with the standards provided to calculate cross-reactivity (Table 2). Cross-reactivity to almond, not included in the kit list, was 2.38 mg kg^−1^. Moreover, cross-reactivity to pecan nut was 3.04 mg kg^−1^, higher than the 0.82% indicated in the kit manual. The LOD and LOQ values calculated in this work using the standard curve were 2.2 mg kg^−1^ and 3.3 mg kg^−1^, respectively, being slightly higher than those indicated in the kit manual (0.6 mg kg^−1^ and 2 mg kg^−1^, respectively). Thus, the sensitivity of this method is similar to other commercial ELISA techniques for walnut detection in food matrices [19].

**Figure 1:**
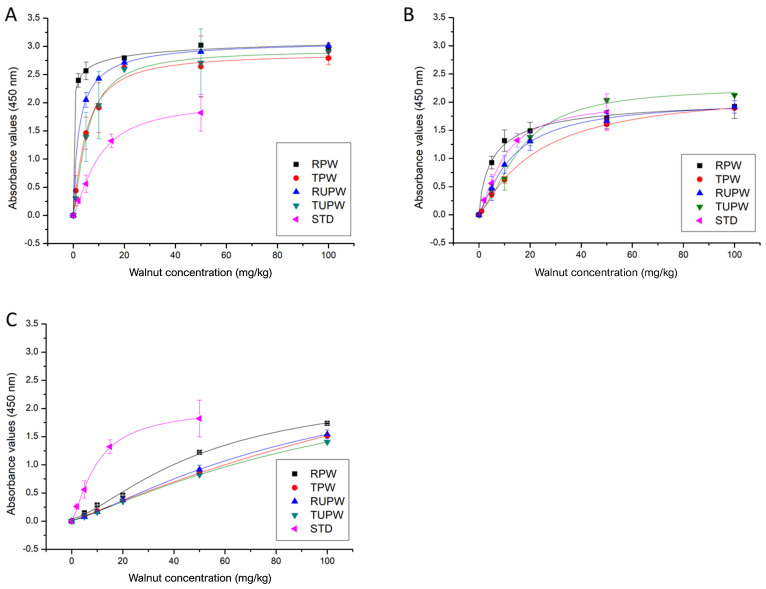
ELISA results obtained from experimental walnut/corn flour binary mixtures prepared with raw peeled walnut (RPW, ■), toasted peeled walnut (TPW, ●), raw unpeeled walnut (RUPW, ▲), and toasted unpeeled walnut (TUPW, ▼) using the Alertox^®^ walnut ELISA kit. The protein extracts from binary mixtures were used undiluted (A) or diluted 1:5 (B) or 1:25 (C) in protein extraction buffer, and results were compared with the standard curve obtained with the ready-to-use standards provided with the kit (STD, ◄). Points represent the mean value ± SD (*n* = 2; 3 independent experiments).

Binary mixtures of walnut in corn flour (100–1 mg kg^−1^) were analyzed following the manufacturer’s directions to evaluate the sensitivity of the kit with food samples. The absorbance values obtained from extracts of the binary mixtures made with peeled and unpeeled walnuts and with raw and toasted walnut (160 °C for 13 min) were plotted against the walnut concentration in the ground mixtures, and compared to the calibration curve obtained using the standards (50, 15, 5, 2, and 0 mg kg^−1^) provided with the kit. The four curves of the experimental mixtures were similar, indicating that the presence of the testa (rich in polyphenols) and toasting [20] do not negatively affect detection of walnut with this kit (Figure 1). 

It should be noted that the absorbance values obtained from the undiluted extracts of the binary mixtures (following the manufacturer’s instructions for sample analysis) were much higher than those obtained for the standard calibration curve (Figure 1A), whilst those obtained with sample extracts diluted 1:5 in the protein extraction buffer were closer to those of the standard calibration curve (Figure 1B). Despite this result, the commercial products were analyzed undiluted to obtain a greater sensitivity to the detection of undeclared walnut, even though quantification would not be reliable. These results reflect that the food matrix could influence the extraction efficiency and absorbance values obtained with the kit.

### 3.2. Evaluation of the Walnut Direct ELISA with Multimeric scFv

The specificity and sensitivity of the multimeric scFv ELISA for the detection of walnut were previously assessed [15]. The LOD was 1616 mg kg^−1^, and some cross-reactivity was found with pecan (2.25%) and almond (0.35%), but not with the remaining tree nuts analyzed. 

**Figure 2:**
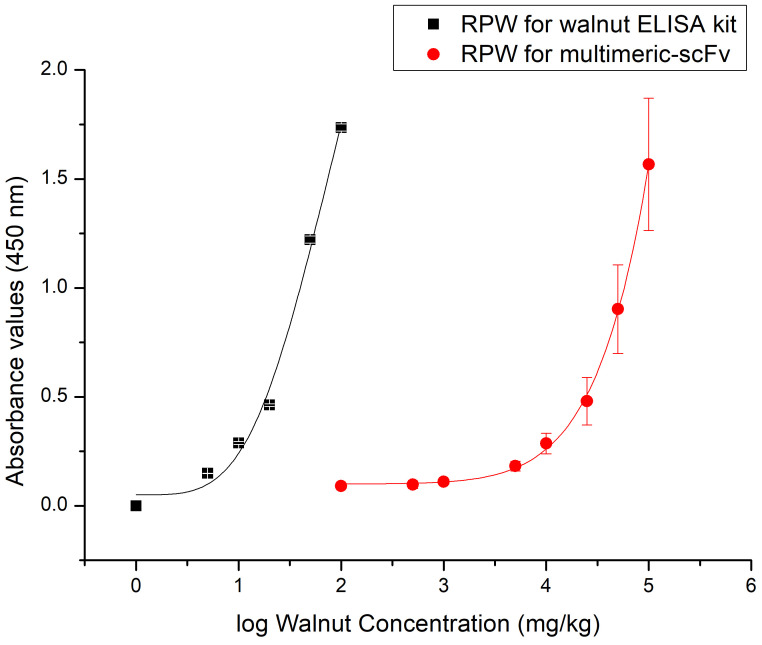
Standard curves obtained from experimental binary mixtures (raw peeled walnut samples in corn flour), analyzed with the Alertox^®^ walnut ELISA kit (■) and the multimeric scFv ELISA (●). Protein extracts were prepared according to directions of each method and diluted 1:25 (ELISA kit) or 1:100 (scFv ELISA) in PBS for comparison. Points represent the mean value ± SD (*n* = 2; 3 independent experiments).

The binary mixtures made with raw peeled walnut in corn flour were analyzed with the walnut ELISA kit and multimeric scFv ELISA to compare performance (Figure 2). The results obtained confirmed that the LOD of the polyclonal antibody ELISA kit is three logarithmical units lower than that of the recombinant antibody-based ELISA.

### 3.3. Identification of the Walnut Proteins Recognized by the JrBSF scFv

**Figure 3:**
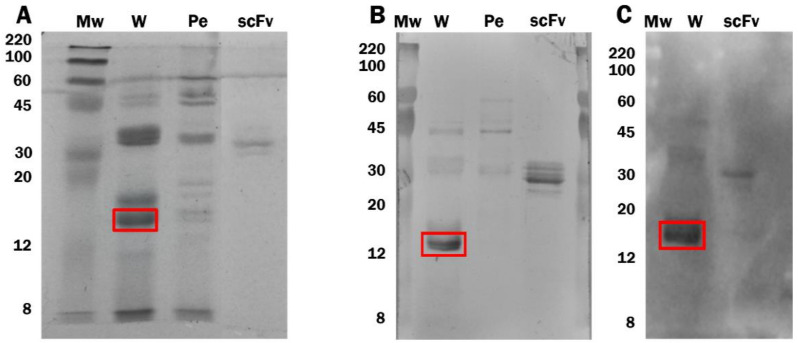
SDS-PAGE electrophoresis (**A**) and JrBSF scFv immunoblots (**B**,**C**) of 10 µg walnut extract (W), 10 µg pecan extract (Pe), and 2 µg purified JrBSF (scFv). Mw of the protein marker bands (ColorBurst^TM^ Electrophoresis Protein Marker, Sigma) is indicated. JrBSF scFv in the immunoblots was detected either with Anti-c-Myc monoclonal antibody (9E10) and alkaline phosphatase (AP) conjugated anti-mouse IgG (**B**) or with horseradish peroxidase (HRP) conjugated Anti-6X His tag^®^ antibody (ab1187) (**C**).

The JrBSF scFv was isolated from a phagemid synthetic library of human scFv fragments (Tomlinson I, Source Bioscience, Nottingham, UK) by phage display against a walnut protein extract [15]. However, the walnut protein fraction recognized by the phage-antibody was not identified. Western blotting analysis demonstrated that a walnut protein band of approximately 15 kDa size was detected by the scFv (square in Figure 3A), as developed with the anti-c-Myc and anti-His tag antibodies (Figure 3B,C). A weak reactivity was also observed in Figure 3B with other higher Mw bands present in both walnut and pecan extracts that corresponded to sizes of approximately 35 kDa and 45 kDa. This result may explain the slight cross-reactivity with pecan observed in the walnut multimeric scFv ELISA.

**Figure 4:**
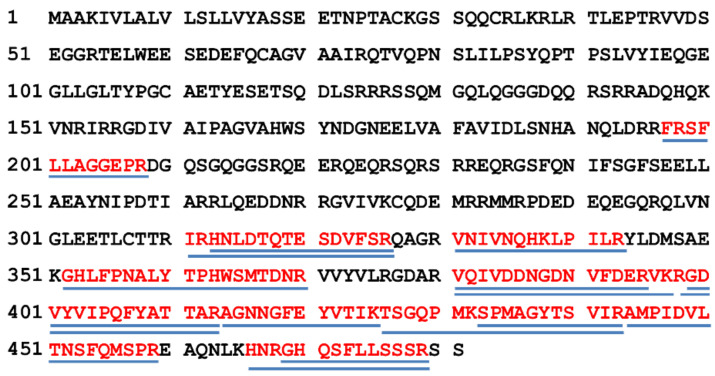
Alignment of peptides identified by MALDI-TOF/TOF tandem mass spectrometry and the MASCOT Database Search engine in the 11S globulin seed storage protein 2-like (*Juglans regia*) (Accession Number: XP_018818401.1).

A proteomic approach was used to confirm the identity of the protein band recognized by the antibody. Nineteen peptides of the 11S globulin seed storage protein 2-like (Jug r 4) from *Juglans regia* (Accession Number: XP_018818401.1) were identified by MALDI-TOF/TOF (Table 3) in the electrophoretic band, with 31% of sequence coverage (Figure 4). 

It is remarkable that the JrBSF scFv specifically recognizes the walnut 11S globulin Jug r 4, which is a relevant minor allergen recognized by 27% of walnut allergic adults and has a predictive value of 90% for walnut allergy [21]. Jug r 4 (11S globulin or legumin) is a high molecular weight protein (approx. 350 kDa) consisting of six subunits forming a hexameric structure that presents an intermediate thermal stability. Intense roasting at 180 °C for 20 min has been reported to increase detection of walnut 11S globulin by LC-MS/MS, possibly due to increased digestibility [22]. However, allergenicity seems to be affected by a great amount of proteolysis and high pressure treatments [20,23].

### 3.4. Analysis of Commercial Products

One hundred samples of commercial products acquired at different stores in Madrid were analyzed, including bakery and pastry products, energy bars, chocolates, ice creams, yoghurts, beverages, sauces, and prepared dishes. The samples were classified into five groups depending on their content of walnut and other nuts as declared on their labelling (Table 4). Only three of the commercial samples analyzed were non-pre-packaged products (a handmade bread and two sandwiches), and they included walnuts in the list of ingredients.

Walnut content was analyzed using the two ELISA methods (direct multimeric scFv and sandwich walnut ELISA kit). In order to avoid false positive ELISA results due to cross-reactions with pecan of the polyclonal (sandwich ELISA kit) and recombinant (multimeric JrBSF scFv) antibodies, the commercial food samples were also analyzed using the previously developed walnut-specific and pecan-specific real time PCR methods [9]. The limit of detection of both real time PCR methods was established at 0.1 mg kg^−1^. Depending on the level of sensitivity of an allergic person (minimal eliciting dose of an individual), exposures to undeclared allergens on products can pose a risk to the food-allergic population. Keeping in mind that it is not possible to accurately assess the amount of allergen that is necessary to elicit an allergic reaction, exposure to 0.03 mg of walnut has been suggested to cause an allergic reaction in one out of every hundred susceptible patients, and 0.08 mg of walnut in 5% of the susceptible patients [24]. According to these eliciting doses, not only the real time PCR technique, but also the walnut ELISA kit and the multimeric scFv ELISA, would be able to detect walnut amounts that could cause allergic reaction to most sensitive consumers (0.01 µg and 0.267 µg walnut per well, respectively) in samples not affected by intense food processing. Nevertheless, in contrast to ELISA techniques, the real time PCR methods are not able to detect or quantify the nut allergens, but rather detect the DNA of the allergenic nut species [8].

Some of the 27 products declaring walnut as an ingredient tested negative for walnut in one or more of the analytical methods used. Walnut was detected in only 17 processed foods by multimeric scFv ELISA, although 26 samples tested positive in the walnut ELISA kit and in the walnut real time PCR. One chocolate sample tested negative for walnut by the three methods (3.7%). The percentage of walnut in the chocolate product (a mousse cream filled chocolate) was not specified, but walnut was declared in the list of ingredients. The protein content of these chocolates is low (4.7 g/100 g) and the epitope recognized by the scFv may have been denatured by chocolate processing [19] or may have formed an aggregate with the polyphenols [25], explaining the negative ELISA results. Considering that the real time PCR method did not detect walnut or pecan, the sample is likely to be mis-labelled. However, chocolate is a food matrix rich in polyphenols that can interfere with analytical methods, and false negatives cannot be completely excluded. 

The nine products that tested negative only in the multimeric scFv ELISA included a sauce (1), beverage (1), yoghurts (5), ice-cream (1), and sandwich (1). This conflicting result could be explained either by the higher LOD of the multimeric scFv ELISA or by denaturation of the epitope recognized by the scFv due to food processing. It should be noted that the manufacture of these products includes at least a pasteurization treatment, or high hydrostatic pressure treatment in the case of the sandwich sample. This effect has been described by other researchers when analyzing the presence of allergens in heat-treated liquid food samples [7,20]. Furthermore, one of the yogurt samples declared to contain 0.1% walnuts (1000 mg kg−^1^), which is lower than the LOD of the multimeric scFv ELISA (1616 mg kg^−1^).

The second group of commercial samples included a precautionary allergen labelling (PAL) regarding walnut content, such as “may contain traces of walnut”. The two samples belonging to this group produced a negative result with the three methods used for analysis, indicating that walnut or traces thereof were not present in these samples. It should be stated that it is difficult to find commercial products that indicate the possibility of containing walnut traces. It is more frequent to find the indication of the possible presence of traces of nuts, as a group, in which this species is also included, but not individually.

Among the 30 products that included one or more tree nut species different than walnut in their ingredients list (Table 4), there were seven out of the ten breakfast cereals that tested positive for walnut by at least one method. All the seven positive samples contained pecan or a mixture of pecan with other nuts, as verified by pecan specific real time PCR. Cross-reactivity with pecan is usually found in all ELISA methods available for walnut detection [23,26]. Therefore, the ELISA methods would be detecting a cross-reaction with pecan. On the other hand, the six samples that tested positive by walnut real time PCR contained around 300 mg kg^−1^ of walnut. According to these PCR results, six of the samples contained both walnut and pecan, while the sample that tested positive only in the walnut ELISA kit contained only pecan and should be considered a false positive for walnut. Therefore, it is not possible to ascertain in these samples whether the ELISA results are detecting walnut and/or pecan. 

From 26 samples labelled as containing traces of other nuts, different than walnut, that were analyzed, three of them belonging to chocolates (3) turned out to be positive. Walnut was detected by PCR in one of the chocolate samples analyzed at a concentration of 230 mg kg^−1^, even though it was negative in the ELISA methods. This may be due to a cross-contamination of the sample, since it was not detected by any immunoenzymatic assay, and the low levels of walnut present in the sample may show unintentional contamination either in raw materials or in the equipment or environment of the food industry. Another reason for not being able to detect walnut in the chocolate samples may be due to the presence of chocolate phenolics that bind walnut proteins and form complexes that inhibit their recognition [25,27]. Among the chocolate samples, two of them were positive by the walnut ELISA kit and real time PCR, so these products were incorrectly labelled.

According to these results, 7.0 to 12.6% of samples (depending on the analytical method) contained walnut but did not declare it, confirming there is a risk for allergic consumers. Similarly, Ford et al. [28] reported almost 3.5% of products positive for allergens did not declare it in the labelling, and 5.3% of products were positive among the “may contain” products analyzed. Allergic consumers show some rejection to those products that state "may contain" in their labelling, either due to distrust in observing the product, because of their knowledge about the manufacturer company, or their past experiences, with up to 8% of allergic consumers reporting having had reactions to the ingestion of PAL products [13]. It is also important to bear in mind that the extensive use of PAL creates frustration for the allergic consumer as it limits the food options to be consumed, while the consumer assumes risks that harm their health [12]. Regulation of the PAL use could improve the safety and quality of life for sensitive consumers. 

Finally, none of the 15 samples “not declaring to contain nuts or nut traces” had a positive result for walnut in any of the techniques described in this study (Table 4).

Analytical methods for detecting allergenic ingredients in foods require adequate specificity and sensitivity to trace very small amounts of the target allergens in complex food matrices and processed foods. The three techniques used in this study have advantages and disadvantages. The real time PCR is the most sensitive of the techniques evaluated, but it does not detect the allergenic protein itself, but rather walnut DNA. Nevertheless, it has been used to confirm both false negative and false positive results because it clearly differentiates walnut and pecan DNA and because of its lower LOD. Despite the higher detection limit of the multimeric scFv ELISA, there was a good agreement between immunoenzymatic and real time PCR results for most commercial samples analyzed, except for some heat-treated liquid samples or those containing pecan. Compared to the multimeric scFv ELISA, the walnut ELISA kit had improved LOD and was less affected by sample processing, but it had a higher cross-reactivity with pecan and other nuts than that stated by the manufacturer, producing false positive results for walnut. On the other hand, in contrast to the walnut ELISA kit, which uses polyclonal antibodies raised in immunized animals, the multimeric scFv ELISA is based on the first recombinant antibody obtained against walnut by phage display, without animal immunization. This is important for fulfilment of international regulations on animal welfare, demanding the avoidance of live animals for scientific purposes whenever possible (European Directive 2010/63/EU) [29]. Moreover, work is currently underway to improve the sensitivity by multimerization on virus-like particles or coupling the scFv to fluorescent molecules that amplify the emission signal for walnut detection. 

## 4. Conclusions

Each method has advantages and limitations for allergen detection, so real time PCR and immunoassays offer complementary approaches [30]. Moreover, food industries must implement good manufacturing practices to elaborate reliable allergen labelling, considering the risk of allergens in their Hazard Analysis Critical Control Points (HACCP) plans. This should minimize the risk of allergic reactions in sensitized consumers.

## Figures and Tables

**Table 1:** DNA sequences and description of the pecan (AF303825) and walnut (HE574850) primers and probes to develop a real time PCR [9].

Detected Species	Primer and Probe	Length (bp)	Sequence (5′ → 3′)	nM	Cycling Conditions
Walnut	WalITSdir	20	GACAATCGGTGGTTGAGAAA	300	Initial denaturation: 10 min 95 °C Amplification: 50 cycles at 95 °C for 5 s 60 °C for 30 s 72 °C for 1 s Cooling: 40 °C for 30 s.
	WallITSinv	20	GTCGAGGAGCACCTTCACAG	900	
	WalITSP	18	6FAM-TGACCCGTCGTGTGTTGCCC-BBQ	2000	
Pecan	PecITSdir	18	ATGAAAGCTGCCCACCGC	300	
	PecITSinv	18	CATTGTTCGACCGGGAAG	900	
	PecITSP	19	6FAM-CGGGTCAGTCTCCTCGTTC-BBQ	2000	
18S	18Sdir	16	TGGTGCCAGCAGCCGC	300	
	18Sinv	25	TCCAACTACGAGCTTTTTAACTGCA	900	
	18SP	22	6FAM-CGCTATTGGAGCTGGAATTACC-BBQ	2000	

The PCR was carried out using the LightCycler^®^ TaqMan^®^ Master (Roche Molecular Systems, Mannheim, Germany) in a total reaction volume of 10 µL in glass capillary tubes.

**Table 2:** Determination of cross-reactivity with heterologous species using the sandwich ELISA kit for walnut.

Sample	ABS 450 nm	SD	Estimated Walnut Concentration (mg kg^−1^)
Walnut	3.055 *	0.112	*
Pecan	0.358	0.019	3.04
Almond	0.274	0.052	2.38
Hazelnut	0.007	0.002	ND
Cashew	0.022	0.003	ND
Pistachio	0.041	0.006	ND
Macadamia nut	0.029	0.015	ND
Brazil nut	0.014	0.011	ND
Peanut	0.055	0.008	ND
Soja	0.008	0.021	ND

ND: Not detected. Below the detection limit using the standard curve of the sandwich ELISA kit (2 mg kg^−1^, A450 = 0.261). *: Undiluted extract of the 100 mg kg^−1^ walnut/corn flour mixture. Above the limits of standard curve. Points represent the mean value ± SD (*n* = 2; 3 independent experiments).

**Table 3:** Peptides identified by MALDI-TOF/TOF tandem mass spectrometry and the MASCOT Database Search engine. Protein scores greater than 83 were significant (*p* < 0.05).

Gel Band	Protein Identification	Accession Number	Sequence Coverage	Total Score	Peptide Sequences
1	11S globulin seed storage protein 2-like [*Juglans regia*]	XP_018818401.1	31%	107	R.FRSFLLAGGEPR.D R.IRHNLDTQTESDVFSR.Q R.HNLDTQTESDVFSR.Q R.VNIVNQHKLPILR.Y K.GHLFPNALYTPHWSMTDNR.V R.VQIVDDNGDNVFDER.V R.VQIVDDNGDNVFDERVKK.R K.RGDVYVIPQFYATTAR.A R.GDVYVIPQFYATTAR.A R.AGNNGFEYVTIK.T K.TSGQPMKSPMAGYTSVIR.A K.TSGQPMKSPMAGYTSVIR.A K.TSGQPMKSPMAGYTSVIR.A K.SPMAGYTSVIR.A R.AMPIDVLTNSFQMSPR.E R.AMPIDVLTNSFQMSPR.E K.HNRGHQSFLLSSSR.S R.GHQSFLLSSSR.S

**Table 4:** Determination of the presence of walnut in commercially processed food products using multimeric scFv ELISA, Alertox^®^ Walnut ELISA kit, and real time PCR.

Label Statement	Product	Number of Samples	Multimeric scFv ELISA	Walnut Kit Alertox^®^	ITS Real Time PCR
**Walnut declared as ingredient**	Biscuit Nut bar Breakfast cereals Chocolate Sauce Bread Beverage Ice cream Snack Yoghurt Sandwich	5 5 1 2 1 3 1 1 1 5 2	+(5) +(5) +(1) +(1)/−(1) −(1) +(3) −(1) −(1) +(1) −(5) +(1)/−(1)	+(5) +(5) +(1) +(1)/−(1) +(1) +(3) +(1) +(1) +(1) +(5) +(2)	+(5) +(5) +(1) +(1)/−(1) +(1) +(3) +(1) +(1) +(1) +(5) +(2)
**May contain** **walnut**	Chocolate Yoghurt	1 1	−(1) −(1)	−(1) −(1)	−(1) −(1)
**Contains other tree nuts**	Biscuit Nut bar Breakfast cereals Chocolate Breadstick Beverage Ice cream	4 9 10 2 1 2 2	−(4) −(9) +(5)/−(5) −(2) −(1) −(2) −(2)	−(4) −(9) +(7)/−(3) −(2) −(1) −(2) −(2)	−(4) −(9) +(6)/−(4) −(2) −(1) −(2) −(2)
**May contain tree nuts traces**	Biscuit Nut bar Breakfast cereals Chocolate Beverage Ice cream	6 5 3 10 1 1	−(6) −(5) −(3) −(10) −(1) −(1)	−(6) −(5) −(3) +(2)/- (8) −(1) −(1)	−(6) −(5) −(3) +(3)/−(7) −(1) −(1)
**Not declaring nuts or traces as ingredient**	Biscuit Nut bar Breakfast cereals Chocolate Sauce Bread Beverage Ice cream Snack	3 1 3 1 1 1 2 2 1	−(3) −(1) −(3) −(1) −(1) −(1) −(2) −(2) −(1)	−(3) −(1) −(3) −(1) −(1) −(1) −(2) −(2) −(1)	−(3) −(1) −(3) −(1) −(1) −(1) −(2) −(2) −(1)

Commercial food products showing estimated walnut concentration lower than the limit of detection (LOD) in ELISA methods, or below 0.1 mg kg^−1^ in PCR, were considered negative. The number in the basket is the number of samples.

## Data Availability

The data presented in this study are available on request from the corresponding author.
